# A Danger at the Soda Fountains

**Published:** 1911-07

**Authors:** 


### A Danger at the Soda Fountains.

With the coming summer the soda fountain with its variety of "soft" drinks and delicacies offers many attractions to young and old.

Quite aside from the questionable use of alcoholic drinks in the flavoring of wine sauces, frozen pudding, etc., served by many of the ice-cream-soda-fountain combinations, another peril has grown up unperceived for the most part which threatens even more formidable dangers—the increasing use of drinks containing caffeine and especially cocaine.

Many dealers serve various bromides also which can not be considered harmless, and their use on lay administration should certainly be discouraged.

The growth of the cocaine habit, the multiplication of the drinks containing these dangerous drugs require that no time be lost in warning the public and especially young people of the dangers. The *Journal*, therefore, brings together data concerning the drinks themselves, the pathological effect of cocaine and caffeine, and the consequences of the easily-formed cocaine habit. Some of the drinks described by Dr. Kebler "contained both caffeine and extract of coca leaf." * * *

Others contained caffeine but there was no evidence that coca leaf in any form had been used in their manufacture. A list of these drinks will be found in Dr. Kebler's article in the report (1909) of the Homes Commission appointed by President Roosevelt.—*Scientific Temperance Journal,* Boston.

